# Miscellany

**Published:** 1887-10

**Authors:** 


### Miscellany.

### *WHAT PROMINENT MEN SAY OF THE CONGRESS.*

Dr. N. S. Davis said to a representative of *The Medical News* on Thursday evening, in reply to the enquiry whether he thought the Congress had been a success, that, in point of numbers, it stood second, if not first.

"But as to its character and the scientific value of its work?"

"Well, I have seen all the presidents of sections to-day, and they each and every one of them report most favorably."

"From your experience of previous congresses, do you think there was a conspicuous absence of distinguished foreigners—such men, for instance, as Paget and Gull, and Pasteur and Verneuil, and Virchow and Lister, and others whose names are known all over the world?"

"Yes, that is so; but then you must remember that many of those you name are over seventy years of age, and at their time of life, and with their professional duties, a journey across the Atlantic, and such a prolonged absence as is thereby implied, is a very serious matter. Sir James Paget himself told me that he feared he would be unable to attend on account of his advancing years. Professor Virchow wrote me that he could not come, because of an important conference to be held in Berlin this month, of which he is chairman of the committee of arrangements. I do not remember exactly what that conference was, but it was something combining hygiene with some other subject."

"But what about Neudörfer and Lutaud, of Paris, who were to have been here and to have taken part in the proceedings?"

"Neudörfer is detained by sickness; and Lutaud—well, I can't say anything about him."

"Then, on the whole, do you think the delegates compare favorably with those of other congresses?"

"Yes, I think so. They are younger men, true, but I understand some very valuable papers have been contributed even by those who did not come. M. Simon, for instance, a very eminent man I am told, has forwarded a most important paper. And you must remember we have had difficulties with regard to the foreign delegates."

"What were those?"

"I do not care to go into them, but, gentlemen—you could count them on the fingers of your two hands—have written and begged foreign delegates not to come. They said there were factional differences. They even went so far as to say that Washington was unhealthy; that the weather was very hot, and that typhoid fever was prevailing. Of course, this kept some away."

"How do you think the American reputation for hospitality has been maintained?"

"That I would rather say nothing about. I have heard many complaints from the foreign delegates that they were not able to get into the White House to see the President and Mrs. Cleveland last night—many such complaints. It was unfortunate, very. I am very sorry it occurred."

"Is there any prospect, do you think, that the Congress will ever meet in the United States again?"

"Not for twenty years at least. These sessions are only triennial, and it is not likely that Washington's turn will come round again for twenty years—if then."

Secretary-General John B. Hamilton was asked how many delegates have registered.

"Over 2,600."

"How many foreigners?"

"Three hundred, it is estimated, but I have not the local registration-book at hand."

"Have you been cramped for want of money?"

"Yes."

"If the dissensions beginning at New Orleans had not taken place, do you think there would have been a larger contingent of foreign delegates?"

"If there had been no system of general misrepresentation practiced by many of those who resigned, and followed up by personal solicitations to stay away, the attendance might have been larger; as it is, the attendance from transatlantic countries is larger than it was at former congresses."

"Do you know of any dissatisfaction having been expressed by foreign delegates as to their treatment here?"

"No, on the contrary; see Sir James Grant's speech to-day on this point."

"What do you think of the scientific aspects of the Congress?"

"They are entirely satisfactory, and will take rank with any former congress. I can only view with contempt the statements made with evident intent to disgrace America and belittle our guests."

"What about the absence of those American physicians who withdrew from the Congress and remained away?"

"While their absence is to be regretted, yet the facts show the Congress is entirely successful without them."

Dr. Unna, of Hamburg, being asked "What opinion he had formed of the Congress?" replied:

"I do not think it is of as high a character as those of Copenhagen and London. The discord at home has prevented many foreigners from coming. This discord was so prominently heralded abroad that it became in a measure international. If it had not occurred, many Germans would have come, not thirty or fifty, but 200. This number would have included the most prominent men of the country. I regret that the leading physicians of America did not overlook the discords when it was too late to reconsider action, and so give to the Congress more of the air of being international. Men like Agnew, Loomis and others are much missed. My strongest impulse in accepting the vice-presidency, and reading a paper, was to prevent the Congress from dwindling into simply a national affair. I regret that others did not take the same view. The scientific aspect, although not equal to former congresses, is better than was to be expected under the circumstances. Papers in the dermatological section were good for the most part, but the discussions were poor; but such is the case at all congresses. Socially, I have not had much personally to complain of, although I am aware of many deficiencies. I know that many errors have been committed. The arrangements have been poorly announced. On the whole, there is much that is to be regretted."

Dr. W. T. Lusk, of New York, was asked what opinion he had formed of the Congress.

He replied: "I have heard some complaints from foreign delegates regarding a want of courtesy on the part of the officers of the Congress. It seems there has been some want of business management, and, perhaps, a lack of experience on the part of the officials in managing such affairs. They have been too much disposed to regard the foreigners as they would our own people, who are always willing to look after themselves, while a European seems to require some one to take care of him."

"What do you think of the scientific value of the Congress?"

"I believe the volumes of transactions, when published, will be equal in respect of the papers contained therein to those of any preceding congress."

"If the unfortunate dissension which began at New Orleans had not occurred, would not the success of the Congress have been more marked?"

"Well, I must say the presence of prominent members of the profession at the discussions was greatly missed."

Dr. Lusk, who was somewhat unwilling to give his views for publication, also stated that he thought that in the division of papers between the sections of obstetrics and gynecology, the former had been unfairly treated by being deprived of several papers properly belonging to it. For instance, a paper on Cæsarean section, and another on extra-uterine pregnancy, had both gone to gynecology, instead of to the proper section—obstetrics."

Dr. Marino Semmola, Professor of Therapeutics in the University of Naples, Italy, was asked his opinion of the present Congress, and replied to the queries addressed to him as follows:

"You have attended other congresses?"

"Yes, indeed, I think I have attended every one."

"What opinion have you formed of the scientific value of the Congress?"

"It is considerably below the average of any of its predecessors?"

"Do you think that if the unfortunate dissensions at New Orleans had not taken place, that a larger number of distinguished men from Europe would have attended this Congress—such men as Porro, from your own country, and Pasteur, Virchow, Esmarch, Paget, MacCormac, etc.?"

"I have met many, if not all those gentlemen, at preceding congresses, and have no doubt at all that if there had been no quarrel among the profession here, some, if not all of them, would have crossed the Atlantic."

"How did you happen to come yourself?"

"I was assured that the quarrel was a matter that had entirely passed over, and so I came on."

"Have you met many of the Americans whom you expected to see?"

"Very few, indeed, and it is a matter of sincere regret to me?"

"How have the social aspects of the Congress appeared to you?"

Dr. Semmola smiled at the question, shrugged his shoulders, and said: "Well, I have had one or two private invitations, but as for the rest—it does not exist."

"You are not favorably impressed, then?"

"No; how can I be?"

---

NOTES AND GOSSIP ABOUT THE INTERNATIONAL MEDICAL CONGRESS.

The total registration of members of the Congress up to Wednesday was a little rising 2,500, including about 110 foreigners. As the registration is carried on by letter as well as personally, this is not an exact representation of the actual attendance. Making due allowance for this fact, it is probable that the real attendance is something over 2,000. There are some noteworthy facts in this regard. There are very few New England physicians, a very small representation from New York and only a part of the Philadelphia men. Some of the Canadian representatives, naturally more familiar with eastern men, lament the absence of many whom they expected and were especially desirous to see. The foreign delegation includes not many men of note. Among the best known are: Dr. Leon Le Fort, of Paris, surgeon at the Hôpital Necker; Prof. Unna, of Hamburg, Germany, editor of the *Journal of Dermatology*; Dr. Joseph Kaposi, of Buda-Pesth, Hungary, director of the Hungarian Bureau of Medical Statistics; Dr. T. Graily Hewitt, of London, the well-known gynæcologist; Dr. Mariano Semmola, of Naples, Italy, a senator in the Italian Parliament; Dr. Apostoli, of Paris; Grant Bey, of Cairo, Egypt; Dr. A. Martin, of Berlin.

Among the exercises of the first day was the election of an enormous list of vice-presidents, prepared for the occasion, most of whom were probably at home. A delegate made a courageous attempt to interrupt the cut-and-dried programme, and to confine the officers to those in actual attendance, but he was promptly and effectually sat upon by the president.

The disadvantages attending the assemblage of a large body of men in a Democratic city, without proper arrangements, have been markedly illustrated. In the first place, it is stated in the Washington papers that there were between 4,000 and 5,000 people at the gathering at the Pension Hall on Monday evening. As the total registration at that time did not exceed 2,000, it may easily be computed how large was the proportion of outsiders. The next night, at the President's reception, the same spectacle was seen of non-members of the Congress thrusting themselves into a place not designed for them. This was carried to such an extent that many of the foreign guests came and went without seeing the President, unable to obtain entrance without long waiting. The upshot has been, that it was announced at the session of Wednesday that it was feared that tickets to the banquet of Thursday had been improperly obtained; and it was, therefore, decided to cancel them and issue new ones.

There is a wide difference in the programmes, and interest of the various sections, and the attendance upon them. For instance, the section devoted to military surgery runs over with papers, and has a definite programme, which is carried out so far as time will allow. The sections on general surgery, gynæcology, diseases of children, and others, perhaps, are well attended and evoke spirited discussions. The section on general medicine is pretty much a failure. There is no regular programme, the promised papers are few, and those that materialize are fewer yet. The morning sessions of this section have been given up for lack of papers and attendance. This may partly be attributed, perhaps, to an essential lack of interest in the subject itself. As a Canadian physician put it, "We are all now surgeons or specialists; we stick to the rectum or what not,—we are no longer doctors."

---

THE *Boston Medical and Surgical Journal* says: "A reasonable verdict as to the Congress seems to be that it was, consideriug the circumstances, fairly successful, and no more. The gentlemen who came from abroad had a right to expect to come to a representative American gathering; and this they failed to find. Sectional, as regards residence, scant in men of recognized professional eminence, the American contingent of the Congress was a body of very worthy family doctors. The whole registration, by the Secretary-General's count, was 2,700, which means an actual attendance of between 2,000 and 2,500. The number of foreign members was not far from 150; and of these there were but few of wide fame. As to the papers presented, quantity, not quality, seems to have been the rule. Instead of the two papers a day before the general session, which should have been the marked features before the Congress, lack of material reduced them to one daily, and of these, one, in the author's own words, was written merely to be read before a section. The address of the President of the Congress fell heavily upon the listening ear. There were, doubtless, among the sectional papers some that were worthy of preservation. Time alone will sift the big wad of documents which ambitious young men of inexperience combined with respectable practitioners to accumulate; but it is believed that the world would weep at the loss of few of them. There inheres, of necessity, in every gathering of the sort a wonderful power of attraction for cranks and wind-beaters; and there was no lack of these among the authors of papers or among those who discussed them. In certain sections, like that on gynæcology for example, where were present eminent men from abroad, (attracted, it may be, by the meeting the succeeding week of the American Gynæcological Society, to which they had been specially invited,) discussion meant more than mere words. But this could not be said in all cases.

As a means for the advancement of science, the success of the Congress was questionable.

So far as the local arrangements were concerned, the job appeared to be too big for the committee in certain directions; at least, the chairman of the committee expressed publicly his dissatisfaction with his own arrangements in as strong terms as did any mere member of the Congress.

The charm of the weather, the beauty of the city, the courtesy shown the Congress by the Chief Executive of the country, all favored the gathering. What it might have been under proper auspices, can be imagined.

